# Research Progress on Saccharide Molecule Detection Based on Nanopores

**DOI:** 10.3390/s24165442

**Published:** 2024-08-22

**Authors:** Bohua Yin, Wanyi Xie, Shaoxi Fang, Shixuan He, Wenhao Ma, Liyuan Liang, Yajie Yin, Daming Zhou, Zuobin Wang, Deqiang Wang

**Affiliations:** 1 International Research Centre for Nano Handling and Manufacturing of China, Changchun University of Science and Technology, Changchun 130022, China; yinbohua@cigit.ac.cn (B.Y.); wangz@cust.edu.cn (Z.W.); 2 Chongqing Key Laboratory of Multi-Scale Manufacturing Technology, Chongqing Institute of Green and Intelligent Technology, Chongqing 400714, China; 3 Key Laboratory for Biorheological Science and Technology of Ministry of Education, Bioengineering College of Chongqing University, Chongqing 400714, China

**Keywords:** nanopore, saccharide molecules, sensor, resistive pulse, ion current rectification

## Abstract

Saccharides, being one of the fundamental molecules of life, play essential roles in the physiological and pathological functions of cells. However, their intricate structures pose challenges for detection. Nanopore technology, with its high sensitivity and capability for single-molecule-level analysis, has revolutionized the identification and structural analysis of saccharide molecules. This review focuses on recent advancements in nanopore technology for carbohydrate detection, presenting an array of methods that leverage the molecular complexity of saccharides. Biological nanopore techniques utilize specific protein binding or pore modifications to trigger typical resistive pulses, enabling the high-sensitivity detection of monosaccharides and oligosaccharides. In solid-state nanopore sensing, boronic acid modification and pH gating mechanisms are employed for the specific recognition and quantitative analysis of polysaccharides. The integration of artificial intelligence algorithms can further enhance the accuracy and reliability of analyses. Serving as a crucial tool in carbohydrate detection, we foresee significant potential in the application of nanopore technology for the detection of carbohydrate molecules in disease diagnosis, drug screening, and biosensing, fostering innovative progress in related research domains.

## 1. Introduction

Carbohydrates, also referred to as saccharides, represent one of the fundamental molecules essential for life [1]. Playing a pivotal role in energy storage within biological systems, carbohydrates typically make up around 15% of plant matter and approximately 1% in animal tissue, serving as the primary energy source for various cellular functions [2]. Moreover, they form the primary constituents of cellulose and pectin, contributing to the structural integrity of cells. Furthermore, carbohydrates are vital for the physiological and pathological functions of cells [3]. The diverse functions of carbohydrates arise from their structural complexity, characterized by intricate molecular mechanisms that are not yet fully elucidated [4,5]. In biological research, the detection of carbohydrates is crucial for studying the growth, development, and metabolic processes of organisms. The presence of carbohydrates is essential for maintaining normal physiological functions of organisms. In the field of medicine, the detection of carbohydrates is of great significance for disease diagnosis and treatment. For example, the detection of carbohydrate levels can help to assess the risk of metabolic diseases such as diabetes [6,7]. In the field of chemistry, the detection of carbohydrates is of great significance for studying their chemical structure and properties. Carbohydrates are still an important source of food and medicine. Through chemical analysis methods, the specific types and structures of carbohydrates can be determined, which is of great significance for fields such as drug development and food processing [8]. Carbohydrate detection plays a crucial role in chemistry, medicine, and biology, demonstrating its irreplaceable value from basic research to practical applications. 

Carbohydrate molecules can be broadly classified into monosaccharides, oligosaccharides, and polysaccharides based on their structural complexity [9]. Monosaccharides are the simplest form of carbohydrates [10], characterized as small-molecule linear aldehydes or ketones, possessing hydroxyl groups on each carbon atom, except those involved in forming aldehyde or ketone functional groups [11]. Serving as the foundational units for the construction of oligosaccharides and polysaccharides, monosaccharides adhere to the empirical formula (C_x_ (H_2_O)_n_) [12]. They can be further categorized based on the position of the carbonyl group, the quantity of carbon atoms present, and chirality [13]. Glucose and fructose are among the most prevalent hexoses in nature, while mannose, galactose, xylose, and arabinose also hold significance as monosaccharides [14]. Oligosaccharides, or oligos, consist of polymers formed by linking monosaccharides via glycosidic bonds, contributing significantly to cell–cell recognition processes [15]. Disaccharides, like sucrose, lactose, and maltose, represent the simplest forms of oligosaccharides. The diversity and intricacy of carbohydrate structures stem from the various manners in which these sugar monomers are connected (α or β bonds) and the specific locations of these connections. Polysaccharides are polymers comprising more than ten monosaccharide or disaccharide units, exhibiting structures that can either be linear or highly branched [16]. Starch, glycogen, cellulose, and chitin are common homopolysaccharides that play crucial roles in cellular physiological and pathological functions [5,17].

The analysis of carbohydrate molecule structures is fundamental to glycochemistry research, with the aim of uncovering the structure–activity relationships of polysaccharides [18,19]. Assessing the structure of saccharides is a highly intricate process, requiring a diverse array of analytical techniques and detection methods. Typically, polysaccharides undergo degradation into monosaccharides using chemical techniques to identify the specific type of monosaccharide present. By integrating instrumental analysis methods such as ultraviolet spectroscopy, infrared spectroscopy, gas chromatography, liquid chromatography, electrophoresis, mass spectrometry, and nuclear magnetic resonance, researchers can determine glycosidic bond types and saccharide configurations, combining multiple methods to gain a comprehensive understanding of polysaccharide structures [20,21,22,23,24,25]. As the comprehension of polysaccharide structure–activity relationships advances, the investigation into chemical modifications of saccharides becomes imperative, aiding in discerning the connection between polysaccharide structures and biological activity. In this context, infrared spectroscopy (IR) and nuclear magnetic resonance (NMR) play vital roles in analyzing the substitution of carbonyl groups within polysaccharide chains and detecting cumulative signals, respectively, despite the latter’s resolution limitations. In addition, the sample demand for IR and NMR technology for the detection of polysaccharides is high, and they are not friendly to polysaccharide samples that are difficult to purify and prepare. The sample preparation requirements are high, for example, for infrared detection, where the sample needs to be kept dry, and for nuclear magnetic detection, the sample needs to be as pure as possible and soluble in deuterated reagents. These methods analyze the overall average structural information of polysaccharides [13,14,26,27].

**Figure 1:**
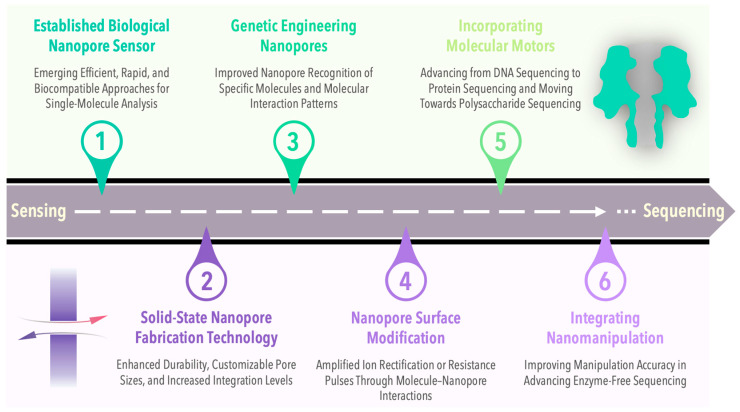
The roadmap presents the vital developmental milestones of nanopore sensing technology. Emerging as single-molecule sensors, nanopores have persistently enhanced their detection sensitivity and range of applications, aiming to realize the sequencing of fundamental biological components like nucleic acids, proteins, and polysaccharides.

Hence, researchers are increasingly eager to acquire structural information on saccharide molecules through direct detection to avoid inaccuracies in polysaccharide structural details resulting from complex detection methods. Over the past few years, with the advancements in nanotechnology, the field of single-molecule detection technology has rapidly gained prominence. K. Kern’s group achieved the visual detection of polysaccharides through mass-selective soft landing electrospray ion separation, polysaccharide molecular beam deposition, and low-temperature scanning tunneling microscope imaging, enabling the discrimination of polysaccharide isomers [28,29]. Peiming Zhang and colleagues demonstrated the capability of recognition tunneling (RT) technology in identifying saccharide molecule isomers at the single-molecule level [9]. Nanopore sensing technology represents a high-throughput, amplification-free, label-free single-molecule detection approach. The utilization of nanopore single-molecule detection technology for the recognition and structural analysis of carbohydrate compounds is emerging as a highly promising new avenue. Figure 1 illustrates the evolution of nanopores from their inception to the present day. Originally conceived as a simple yet efficient single-molecule sensor, they have evolved into a growing investigative tool for deciphering the mysteries inherent in the fundamental components of life’s origins. Nanopores are primarily categorized as biological nanopores and solid-state nanopores [30]. Biological nanopores are created through the self-assembly of biomolecules, such as proteins, in lipid bilayers [31,32,33]. These nanopores have the capacity to identify and detect molecules with diameters ranging from approximately 1 to 10 nm [34]. On the other hand, solid-state nanopores are nanoscale apertures engineered on synthetic thin films, featuring customizable pore sizes and positions, alongside exceptional thermal and chemical stability in their structure [35,36]. These nanopores can be scaled from a few nanometers up to several hundred nanometers, rendering them suitable for the analysis of large biomolecules or complexes [37,38,39]. Leveraging nanopore sensors, diverse saccharide molecules can be distinguished, offering novel experimental insights into nanopore electrodynamics and unveiling the properties of saccharide molecules. Recently, Yan Zhao et al. provided a review of research on saccharide molecule detection employing various nanopore technology strategies from the perspective of nanopore detection strategies [40]. Additionally, Guangda Yao et al. assessed the current state of nanopore-based polysaccharide sequencing technology and presented future prospects for nanopore polysaccharide sequencing methodologies [41].

**Figure 2:**
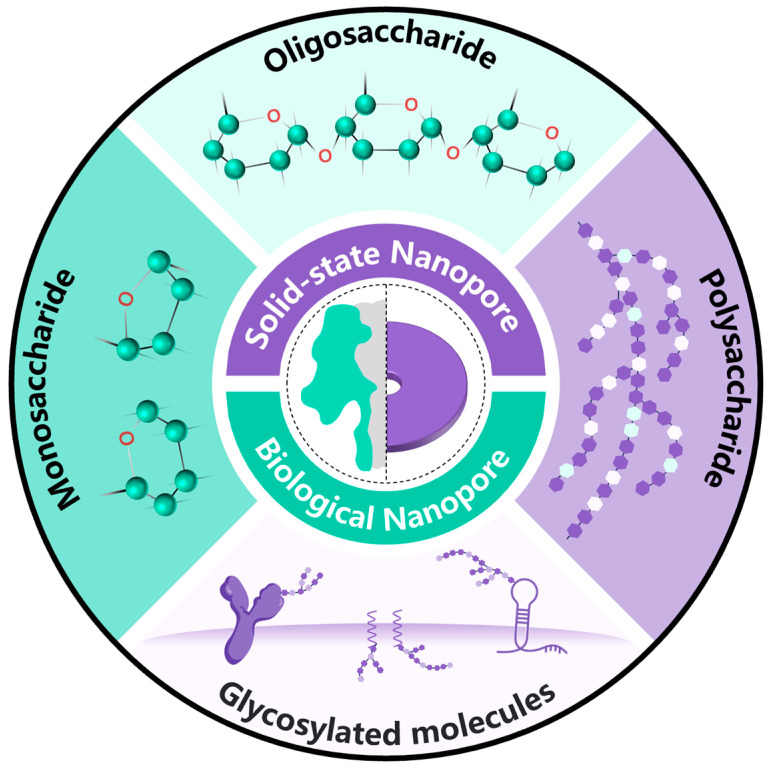
Outlines the potential of nanopore technology for detecting various saccharide molecules. Mainly detecting four types of saccharide molecules including monosaccharides, oligosaccharides, polysaccharides, and glycosylated molecules. Nanopore technology mainly includes two detection techniques: biological nanopores and solid-state nanopores. These two nanopore detection techniques have made certain contributions to the detection of these four types of saccharide molecules.

In this review, the potential of nanopore technology for detecting diverse saccharide molecules is investigated (Figure 2). The main methods for monosaccharide detection involve utilizing the resistance pulse method of biological nanopores or the ion rectification detection technology of solid-state nanopores to achieve high-sensitivity detection of monosaccharides, including glucose. Oligosaccharide detection primarily relies on single-molecule detection technology using biological nanopores, enabling the identification of oligosaccharides with varying degrees of polymerization and the distinction of glycosidic bond connection modes. Solid-state nanopore technology is predominantly employed for polysaccharide detection, targeting polysaccharides like heparin and hyaluronic acid. The characteristic signals of resistance pulses are directly analyzed to differentiate between different polysaccharides. These nanopore-based saccharide molecule detection technologies exhibit promising application prospects in disease diagnosis, drug monitoring, food safety, and related fields.

## 2. Monosaccharide Detection Based on Nanopore Technology

**Figure 3:**
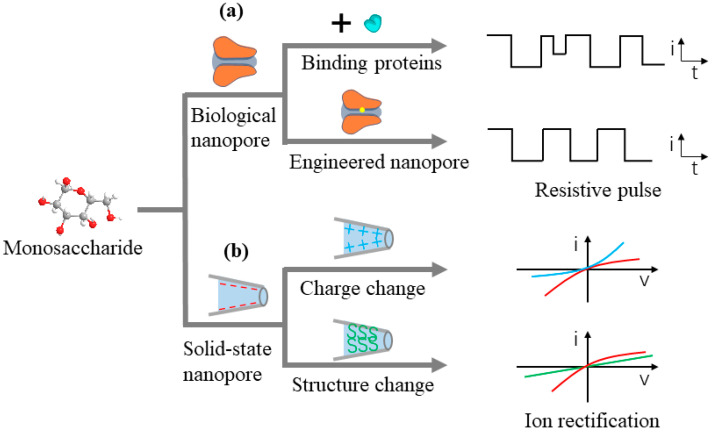
Schematic of monosaccharide detection strategy based on nanopore technology. (**a**) Biological nanopore detection techniques rely on resistive pulse sensing; (**b**) solid-state nanopore detection techniques rely on ion rectification detection strategy (red line is the original rectification behavior; blue line is the charge changed line; green line is the structure behavior behavior.).

Monosaccharides, characterized by relatively small molecular sizes, are subject to high-sensitivity single-molecule detection employing both biological nanopores and solid-state nanopores. Notably, biological nanopore detection techniques rely on resistive pulse sensing to differentiate monosaccharides within mixtures based on distinctive blockade current signals. This methodology represents the predominant sensing approach for nanopores, where the analytes induce a transient increase in pore resistance, leading to a blockage pulse signal. The amplitude and the dwell time correlate with the size of the analyte, while the signal’s shape elucidates the mode of interaction between the analyte and the nanopore. Owing to size restrictions, biological nanopores exhibit limited sensitivity to monosaccharide molecules; however, their blocking behavior can be optimized by utilizing specific binding proteins or incorporating potential interaction sites (Figure 3a).

In contrast, solid-state nanopore detection of monosaccharides hinges on the ion rectification detection strategy. The underlying principle entails the regulation of ion transport properties within the nanopore channel. By applying varying voltage polarities to the nanopore, asymmetry is observed in the ion current. The introduction of different surface-modifying groups on the solid-state nanopore surface, combined with monosaccharide molecules, will modify the surface charge (blue line) or the surface conformation of the nanopore channel (green line), ultimately leading to a shift in the channel’s original rectification behavior (red line) (Figure 3b).

### 2.1. Research on Monosaccharide Detection Based on Biological Nanopores

**Figure 4:**
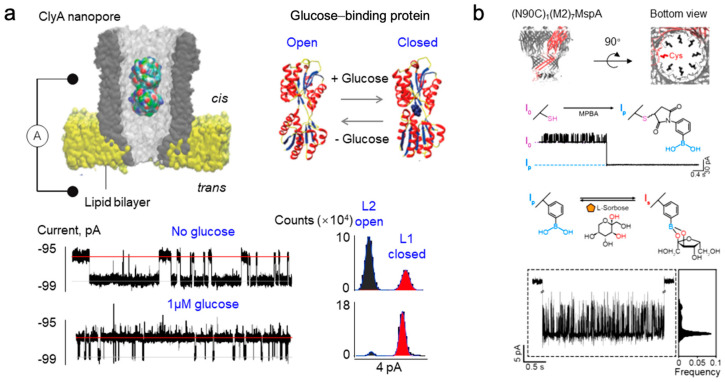
The detection of monosaccharides utilizing biological nanopores is depicted. (**a**) A direct quantitative detection method for glucose in body fluids was developed through the identification of protein conformation by ClyA nanopores and GBP−based nanopore sensing [46]. (**b**) Monosaccharide sensors were established by exploiting boronic acid-modified MspA nanopores and reversible covalent interactions between boronic acid and diols under aqueous conditions [51].

Glucose, a crucial monosaccharide in biological activities [42], not only provides essential energy for human metabolism and organ function but also serves as an important indicator of diseases such as diabetes and hyperglycemia when its levels are abnormally elevated [43]. Presently, two primary strategies for monosaccharide detection based on biological nanopores exist: one utilizes glucose-binding proteins (GBPs) for high-sensitivity detection by leveraging their binding characteristics with monosaccharide molecules, while the other involves modifying biological nanopores to enhance interactions with monosaccharides, thus enabling specific recognition. GBP refers to a type of protein capable of binding specific substrates, demonstrating two conformations: an open conformation in the absence of ligands and a closed conformation when bound to ligands [44,45]. Through monitoring the conformational changes in GBP via the cytolysin A (ClyA) nanopore, glucose in body fluids can be quantitatively detected [46]. Upon adding glucose to the trans side of the nanopore, the blocking events corresponding to the closed conformation of GBP significantly increase, with the blocking frequency displaying a linear correlation with glucose concentration (Figure 4a). The fractional time of GBP in the closed conformation also aligns well with glucose concentration. Furthermore, the quantitative assessment of glucose remains consistent across various sample types; the testing system maintains stability for a specified duration, even with untreated samples like blood, sweat, urine, and saliva. This research was additionally extended to detect the activity of influenza A virus Neuraminidase (NA) [47]. By binding galactose specifically through GBP, the sensor can real-time detect galactose produced from the cleavage of sialic acid-galactose (SG) by influenza virus NA at the single-molecule level. The detection limit for NA is 0.17 ng/mL. Moreover, this sensor can assess the inhibitory effects of small-molecule antiviral drugs like zanamivir on NA activity.

The ClyA nanopore sensor presents a highly sensitive, label-free platform for early influenza diagnosis and rapid antiviral drug screening. The common practice of modifying biological nanopores with boric acid is a key strategy for monosaccharide detection. This method primarily relies on the reversible covalent interaction between boric acid and saccharide molecules containing cis-diol structures under aqueous conditions [48]. Hagan Bayley et al. engineered the α-hemolysin (α-HL) nanopore, comprising a single cysteine mutant T117C and six copies of the wild-type protein (WT)_6_(T117C)_1_, which can be utilized for the single-molecule sensing of monosaccharide isomers [49]. Monitoring the variations in ionic current through the nanopore reveals the formation and dissociation of individual boronic acid–diol bonds that correspond to blockage events, allowing for the identification of the isomeric structure of the saccharide attached to boronic acid within an equilibrium mixture. This research differentiated among D-maltose, D-glucose, and D-fructose components in mixtures, thus advancing the development of more precise glucose monitoring devices for medical diagnostics [50]. The boronic acid-modified Mycobacterium smegmatis porin A sensor, MspA-PBA, enhances the precision of monosaccharide component analysis [51] (Figure 4b). D-fructose, D-galactose, D-mannose, and D-glucose are among the most prevalent monosaccharides in nature, and their identical molecular weights pose a challenge for distinguishing them solely based on the amplitude and frequency of the nanopore blockage current. However, employing machine learning analysis enabled the extraction of multidimensional data from the raw current blockage traces encompassing the mean (ΔI/I_p_), standard deviation, skewness, kurtosis, minimum, maximum, peak-to-peak value, median, and dwell time. This comprehensive approach led to the successful identification of nine monosaccharides, including D-fructose, D-galactose, D-mannose, D-glucose, L-sorbose, D-ribose, D-xylose, L-rhamnose, and N-acetyl-D-galactosamine. It also distinguished subtle structural variations between anomers, achieving an outstanding accuracy score of 0.96. This sensing strategy extends to various saccharide molecules and may yield new insights into nanopore saccharide sequencing.

### 2.2. Monosaccharide Detection Based on Solid-State Nanopores

Solid-state nanopore technology, renowned for its exceptional thermal stability and pH tolerance [52], offers notable advantages in monosaccharide detection, particularly in non-physiological conditions. While small-sized solid-state nanopores pose challenges in reproducibility, the detection of monosaccharides can be effectively achieved by creating large-diameter nanopores and implementing chemical modifications. Phenylboronic acid and its derivatives act as probes for saccharide detection, forming cyclic esters with saccharide molecules [53]. This provides two primary principles for monosaccharide detection: the charge method and the size–structure method (Figure 3b). One method involves adjusting the rectification ratio to achieve high-sensitivity detection through the charge changes resulting from the interaction of phenylboronic acid groups with saccharide molecules. The other method focuses on monitoring changes in ionic current by detecting size–structure alterations induced by the binding of polymer molecules to saccharide molecules. These strategies not only enhance the detection capabilities of solid-state nanopore technology but also pave the way for novel applications in the future.

**Figure 5:**
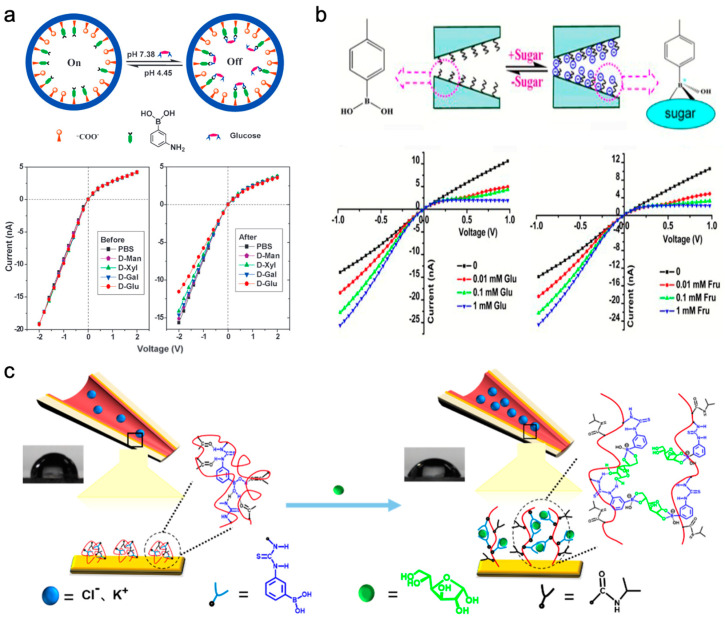
The detection of monosaccharides utilizing solid-state nanopores is depicted. (**a**) pH-gated glucose-responsive biomimetic nanopores engineered using chemically modified nanopores with APBA for glucose detection [56]. (**b**) The P(DMAEMA-*co*-VPBA) copolymer-modified biomimetic nanopores demonstrate rectification responses to pH, temperature, and the presence of sugar molecules [59]. (**c**) By utilizing a polymer modified with Au-S-bonded PATPBA-*co*-PNIPAAm, this nanopore sensor can detect glucose in saliva, prompting alterations in the conformation and wettability of the copolymer film to produce rectification responses [61].

The specific interaction between boronic acid and cis-diol structures allows boronic acid to reversibly react with 1,2- or 1,3-cis-diols to form five- or six-membered cyclic boronic acid esters. Based on the characteristic of cyclic ester opening and releasing cis-diol compounds in acidic environments, pH biomimetic gating can be achieved [54,55]. This phenomenon can give rise to a pH-gated biomimetic configuration in solid-state nanopores [56]. Conical polyethylene terephthalate (PET) nanopores were utilized to introduce 3-aminobenzeneboronic acid (APBA) through the classic EDC/NHS reaction. The resulting modified nanopores demonstrate pH-dependent glucose sensitivity, assessed by monitoring alterations in transmembrane ionic current (Figure 5a). The reduction in negative charges on the channel surface following the reaction of APBA with glucose leads to an increase in the rectification ratio with glucose concentration, serving as an indicator of variations in glucose concentration. Single glass conical nanopore channels modified with 4-carboxyphenylboronic acid (CPBA) can enhance rectification sensing stability [57]. Functioning as a weak Lewis acid, CPBA remains neutral under physiological conditions at pH 7.4 [58]. Upon the addition of glucose, the nanopore device adjusts its ionic transport properties, transitioning from non-rectifying to rectifying states. The rectification ratio of ionic current shows a straightforward linear regression relationship with glucose concentration, with a remarkably low detection limit of 10^−3^ mM. To amplify the intelligence and biomimicry of the nanopore system, P(DMAEMA-*co*-VPBA) copolymer was immobilized on the inner walls of glass conical channels using the surface-initiated atom transfer radical polymerization technique, enabling rectification responses to pH, temperature, and the presence of saccharide molecules [59]. In this context, pH modulates the ion selectivity of the nanopores, temperature influences the conformational alterations of the polymer brush, and the introduction of saccharide molecules further fine-tunes the rectification ratio, showcasing responsiveness to glucose and fructose (Figure 5b).

Similarly, high-polymer molecules containing abundant phenylboronic acid groups can be used to modify nanopores, achieving the high-density modification of phenylboronic acid groups within the nanopores and improving the sensitivity of monosaccharide molecule detection. There are two main ways to modify polymers in nanopores. One is to select positively charged polymer molecules and modify them in negatively charged nanopores based on electrostatic adsorption; one method is to modify the nanopore through covalent bonding. By changing the surface charge or surface wetting state, the ion rectification signal can be altered to achieve a highly sensitive detection of monosaccharide molecular properties. The non-covalent binding of polymers to nanopores presents a straightforward and efficient novel approach for rectification sensing. Through the physical adsorption of poly(vinylpyridine) quaternized with benzylboronic acid onto a quartz nanopipette, precise control over ion current rectification properties is attainable [60]. The modified nanopores exhibit notable cationic rectification features in a pH 9.5 buffer solution. In alkaline conditions, the polymer attaches to saccharide molecules, leading to a swift reversal of current rectification, indicating the polymer’s conversion to a zwitterionic state. In particular, under negative potentials, the presence of fructose substantially amplifies ion current due to the polymer’s affinity. By gauging ion current intensity, a functional relationship correlating to fructose concentration can be established, enabling the quantitative assessment of fructose levels. Moreover, the sensitivity of ion rectification detection for monosaccharides continues to enhance. Remarkably, high-sensitivity glucose detection in saliva has been achieved by employing a polymer modified with Au-S-bonded PATPBA-*co*-PNIPAAm [61]. This copolymer undergoes a wettability transformation in the presence of glucose, inducing fluctuations in ion current and thereby facilitating glucose detection. These investigations have underscored how by modifying the inner surface of solid-state nanopores with specific interactive groups such as phenylboronic acid and its derivatives, precise and selective monitoring of monosaccharide concentrations can be accomplished based on alterations in ion current patterns (Figure 5c). This methodology tracks the binding occurrences of monosaccharides to the modification groups, resulting in changes in the ionic current characteristics of the nanopores, thus enabling the quantitative analysis of saccharide molecules, especially suitable for monitoring monosaccharides in biological specimens like saliva.

## 3. Oligosaccharides Detection Based on Nanopore Technology

Current nanopore sensing techniques for identifying oligosaccharide structures predominantly rely on biological nanopore single-molecule detection technology. The targets for detection encompass linear neutral oligosaccharides like maltose and glucan oligomers, cyclic saccharide molecules, and anionic oligosaccharides such as glycosaminoglycans. Analysis of the current pulse blockade signals reveals the capability of biological nanopore detection technology to discern various polymerization levels in linear neutral oligosaccharides, characterize glycosidic linkage types, differentiate cyclic saccharide molecules, and investigate the dynamic processes of enzymatic reactions.

### 3.1. Research on the Detection of Neutral Oligosaccharides

There are three main principles for detecting neutral oligosaccharide molecules in nanopores: one relies on specific protein nanopores to detect oligosaccharide molecules; the second is based on the specific recognition of protein molecules to assist detection; and the third is to add molecular tags to enhance the charge of oligosaccharide molecules and their interaction with biologic nanopores.

The following regards the detection of neutral oligomaltose: At first, Lisen Kullman et al. used maltoporin to directly detect oligosaccharides of different molecular weights. His research team explored how various sizes of maltose oligosaccharides traverse a planar lipid bilayer subsequent to the reconstitution of maltoporin [62], investigating the transmembrane transport mechanism of saccharide molecules, the interaction between saccharide molecules and proteins, and the molecular conformation of saccharides. Their investigations revealed that all examined saccharide molecules, ranging from maltotriose to maltoheptaose, effectively impeded the ion flow of a monomer within the maltose porin trimer. Of note, maltohexaose exhibited a notably profound blocking effect, capable of obstructing the monomer’s current by a minimum of 98%. The study elucidated the inherent transport mechanism of saccharide molecules, underscoring the potential of single-channel experiments in unraveling the intricate molecular characteristics of saccharides.

**Figure 6:**
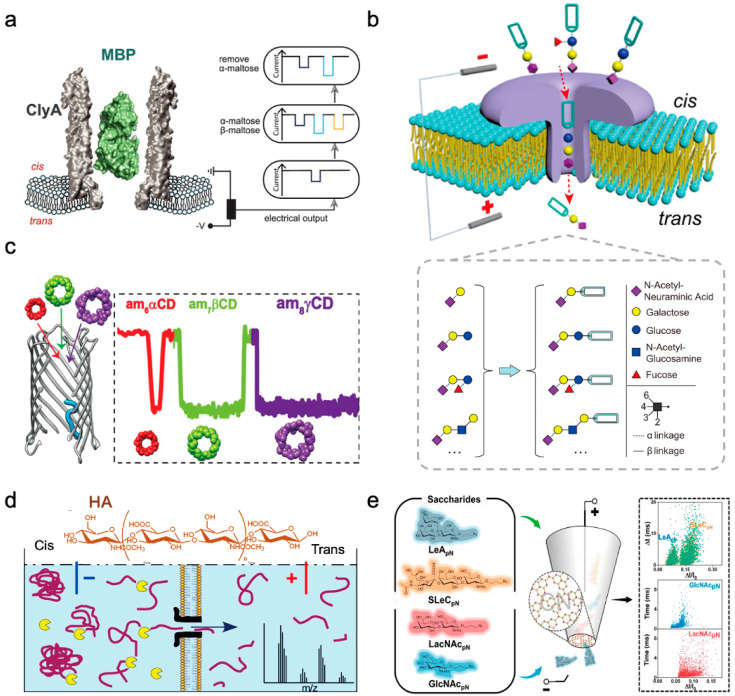
The identification of oligosaccharides using nanopores is illustrated. (**a**) A spatial sensitivity evaluation of diverse isomeric saccharides binding conformations of MBP utilizing ClyA nanopores [63]. (**b**) Employing wild−type Ael nanopores for the direct discrimination of oligosaccharide derivatives subjected to labeled modifications [65]. (**c**) An examination of the distinctive absorption and conveyance of cyclic sugars facilitated by the CymA nanopore [66]. (**d**) An investigation into the pore attributes of glycosaminoglycans hexose, octaose, and decaose employing the AeL protein pore [69]. (**e**) Employing the porous structure of CN to examine two diagnostic trisaccharide epitope derivatives of LeA_pN_ and SLeC_pN_ [72].

The differentiation of oligomeric maltose isomers was achieved using ClyA conventional pore and maltose binding proteins (MBPs). The ClyA nanopores, acting as highly spatially sensitive molecular tweezers, can dynamically track the conformational alterations of individual MBPs in real-time by capturing them (Figure 6a), and subsequently assess their binding interactions with saccharide molecules [63]. Utilizing additional biochemical assays, along with kinetic and thermodynamic evaluations, it was determined that MBP displays three distinct ligand-binding states when exposed to reducing saccharide molecules, depicting MBP’s engagement with diverse α- and β-isomers of oligomaltose. This revelation not only enhances our comprehension of the mechanism governing MBP’s substrate recognition but also underscores the potency of nanopore tweezers as a potent, label-free single-molecule technique for scrutinizing the structural dynamics of proteins in operational settings.

Based on their larger pore size, α-HL nanopores enable the direct detection of the diffusion of oligomaltose and oligoglucose in solution. The α-HL nanopores can readily differentiate oligosaccharides based on their polymerization levels and glycosidic bond types [64]. This study focused specifically on two oligosaccharide varieties: maltose oligosaccharides linked via 1→4 glycosidic bonds and glucan oligosaccharides linked via 1→6 glycosidic bonds. An observable trend emerges showcasing a reduction in the passage frequency of maltose oligosaccharides through the nanopores with increasing molecular weight, whereas glucan oligosaccharides exhibit greater cross-sectional occupancy and elevated hydrodynamic radius. These findings suggest that smaller oligosaccharides can diffuse through the nanopores freely, while larger counterparts face restriction within the pores, compelling an elongated conformation. The discrepant behaviors of glycosidic bonds (1→4 vs. 1→6) during nanopore transport unveil a novel distinguishing approach for discerning between these bond types.

However, the small molecular size and low charge density of oligosaccharide chains present challenges in directly detecting saccharides through nanopores. By employing tags as a practical saccharide chain derivatization strategy, the sensing of oligosaccharide chains was successfully accomplished using wild-type aerolysin (AeL) nanopores [65]. The highly cationic nature of the AeL pore interior and the cation–π interactions with aromatic molecules linked to the oligosaccharide chains enhance their interaction at the nanopore interface, leading to robust nanopore signals. Incorporating substantial labels can notably amplify the distinctions between saccharide chain isomers, facilitating the analysis of their isomeric configurations (Figure 6b). This approach enables discrimination not only among positional and stereoisomers of saccharide chains but also the identification of oligosaccharide chains differing in monosaccharide composition and branching patterns. Furthermore, integrating machine learning techniques can enhance the accuracy of identifying oligosaccharide chains, offering novel avenues for saccharide chain analysis and potential oligosaccharide chain sequencing.

In addition to the determination of linear oligosaccharides, research has also been conducted on the detection of circular oligosaccharides, using specific nanopores (CymA) to achieve the detection of complex cyclic sugar molecules. The bacterial saccharide transporter pore, CymA, facilitates the transport of intricate large cyclic oligosaccharides (Figure 6c). Comparative analysis between natural CymA and a truncated form lacking the N-terminal segment revealed potential residual N-terminal obstructions in the well during substrate transportation, thereby modulating the transport process [66]. The significance of molecular charge, size, and symmetry in enabling transport through CymA pores was underscored in the study. These insights contribute to elucidating how bacteria utilize specific membrane protein pores for molecule transportation. Natural CymA serves as a nanopore sensor enabling the single-molecule detection of sizable cyclic saccharide molecules, achieving the simultaneous detection of complex cyclic saccharide molecules at a single-molecule resolution level. This research paves the way for leveraging nanopore technology in biological detection and molecular transport investigations.

### 3.2. Research on the Detection of Glycosaminoglycans

Glycosaminoglycan molecules have abundant charge, so direct detection is mainly used. The research on glycosaminoglycans mainly focuses on how to focus on more accurate structural information, such as distinguishing glycosidic bonds. or focuses on their biological performance, such as enzymatic hydrolysis.

The advancement of engineering technology in biological nanopores has spurred innovations in saccharide structure analysis. Engineered α-HL nanopores have depicted the topography of acetylamino and carboxyl groups along oligosaccharide chains at mono-, di-, and trisaccharide levels, showcasing the capability of nanopores in directly discerning intricate structural disparities within complex saccharide chains [67]. The modified α-HL nanopore variant M113R/T115A further enhances the detection of complex oligosaccharide chains under micromolar concentrations and label-free circumstances. This investigation delves into oligosaccharides from the poly-N-acetylglucosamine series, highlighting structural variations. By leveraging ion current characteristics, particularly amplitude variance, for three-dimensional profiling, distinctive oligosaccharide features are identified, including variations in functional group quantities, chain lengths spanning from disaccharides to decasaccharides, and diverse glycosidic bonds. This study validates the feasibility of utilizing nanopore sequencing technology for elucidating complex oligosaccharide chains [68].

Nanopore technology offers a versatile approach to analyze the depolymerization of oligosaccharides derived from polysaccharides. The transportation of glycosaminoglycan oligosaccharides was successfully observed using AeL protein pores, marking the inaugural demonstration of hyaluronic acid oligosaccharides translocation via protein nanopores [69]. Notably, the average residence time of oligosaccharides escalates with heightened polymerization degrees (Figure 6d), showing consistency with prior research involving maltose and glucan oligosaccharides. Leveraging the nanopores’ ability to discriminate oligosaccharides at the single-molecule level, this study monitored the real-time catalytic degradation process of high-molecular-weight hyaluronic acid polysaccharides by hyaluronidase. Further investigation entailed kinetics analysis of enzymatic hydrolysis facilitated through nanopores, combining experimental observations and data modeling. Nanopore sensors excel in real-time concentration measurements and evolution monitoring in reaction product size distribution, offering substantial value for tracking diverse enzymatic reactions instantaneously. A comparative study on glycosaminoglycan hyaluronic acid transport through AeL and α-HL nanopores, possessing similar channel dimensions [70], revealed that while AeL nanopores detect hyaluronic acid oligosaccharides effectively, α-HL nanopores exhibit limited detection capabilities. Subsequent experiments highlighted a reversal in outcomes when larger saccharide molecules entered the nanopore from the stem side. Sensing events within α-HL nanopores exhibited a broad range from microseconds to milliseconds; however, those in AeL nanopores clustered within tens to hundreds of microseconds. These findings emphasize that the selection of nanopores for resistance pulse sensing experiments should consider not just the inner diameter but also aspects like charge redistribution within the pore, specific analyte–channel interactions, and channel geometry. Moreover, the transport behavior of polyelectrolyte molecules, specifically dextran sulfate through α-HL nanopores, shows that polyelectrolyte saccharide molecules penetrate the pores only when the Debye shielding length is less than the theoretical pore radius [71]. A higher potential gradient proves more conducive to molecule transport, with the residence time experiencing a pronounced surge with escalating Debye length, particularly nearing the aperture radius.

With the continual refinement of solid-state nanopore assembly techniques, advancements in oligosaccharide structure detection have emerged. Linru Guo et al. successfully differentiated two trisaccharide variants with subtle structural disparities utilizing nanoscale porous thin-film materials and glass nanopores [72]. Carbon nitride (CN) stands out as a contemporary layered material distinguished by its precise pore architecture [73]. The ultra-thin composition of nanosheets guarantees exceptional spatial resolution in nanopore device functionality. The research focused on analyzing two diagnostic trisaccharide epitope derivatives, LeA_pN_ and SLeC_pN_ (Figure 6e). Notably, the robust affinity observed between CN nanopores underscores the significant potential of CN in single-molecule detection. Validation has been achieved through the capability of CN nanopores to discern two carbohydrate epitopes displaying marginal structural distinctions directly. Furthermore, the sensing system exhibited proficiency in discriminating monosaccharides and disaccharides, highlighting the resilience and promise of CN nanopores in spatial resolution of monosaccharides. CN nanopores are thin-film materials with uniformly sized nanoscale pores prepared by chemical synthesis methods, which are a breakthrough in the preparation technology of solid-state nanopores. This chemical synthesis technique significantly improves the uniformity of small-pore-size nanopore preparation with atomic-level precision. However, this material has a high pore density, which increases the difficulty of analyzing nanopore signals.

The above research indicates that nanopore detection technology provides an effective oligosaccharide characterization method for simultaneously detecting the conformation and size of oligosaccharides, which is a new direction for the future development of polysaccharide structure detection. The construction of new nanopores also lays the foundation for the direct differentiation of oligosaccharide structures.

## 4. Polysaccharide Detection Based on Nanopore Technology

The complexity arising from variations in composition, size, charges, and isomeric forms of polysaccharide structures necessitates the predominant use of solid-state nanopores for polysaccharide detection methods. For polysaccharides with good linearity, biological nanopores have also been used for detection research. Solid-state nanopore technology includes the detection of more types of polysaccharides. These methods encompass the analysis of high-molecular-weight polyanionic polysaccharides like heparin and hyaluronic acid. The differentiation of polysaccharides with comparable structures is achieved through the distinct analysis of resistance pulse signals and machine learning algorithms. Leveraging the size-dependent detection capabilities of polysaccharides, our study directly measured enzyme reaction durations at the single-molecule level. Furthermore, our research team delved into the exploration of plant polysaccharides utilizing nanopore technology, affirming the adeptness of solid-state nanopores in detecting and characterizing polysaccharide structures.

### 4.1. Research on the Detection of Heparin Using Nanopore Technology

Heparin (HEP) is a high-molecular-weight glycosaminoglycan, and direct detection based on solid-state nanopores is mainly used for heparin detection. HEP serves as a prevalent anticoagulant for addressing various coagulation disorders. In the past decade, the contamination of heparin due to chondroitin sulfate (OSCS) has emerged as a critical health concern [74]. Therefore, the current focus is mainly on how to combine algorithms to more accurately distinguish heparin and its analogs.

**Figure 7:**
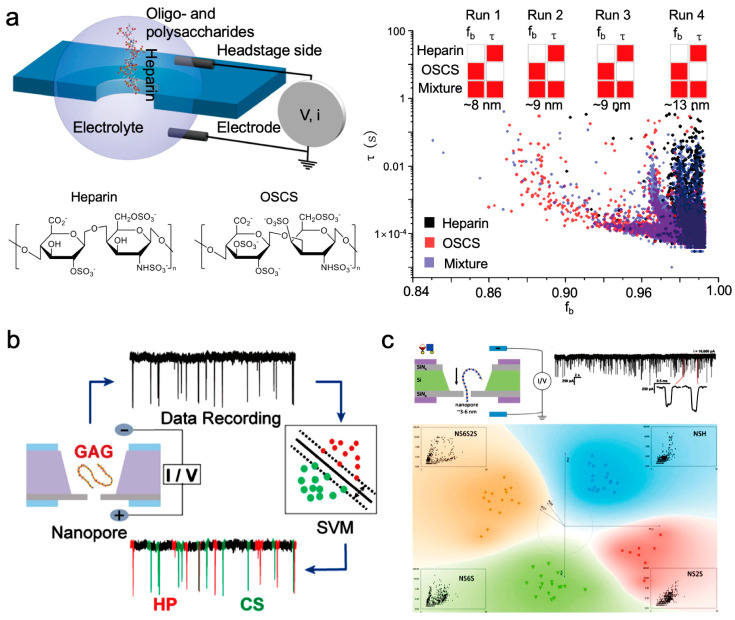
The detection of heparin-like polysaccharides using solid-state nanopores. (**a**) Variations in event features’ distribution between heparin and OSCS samples. A solid-state silicon nitride nanopore sensor was employed for direct analysis of heparin and other polysaccharides using a straightforward statistical threshold algorithm [75]. (**b**) The utilization of a solid-state nanopore single-molecule sensor and SVM to develop a machine learning algorithm for the precise quantification and identification of GAG [76]. (**c**) Polysaccharide nanopore event data, portrayed as a scatter plot, were transformed into feature vectors, subsequently compressed using PCA for effective classification and recognition [78].

Dwyer’s research team introduced the utilization of thin-film solid-state nanopore sensors and threshold algorithms to elucidate polysaccharide properties. The endeavor to explore silicon nitride nanopores as detection tools for polysaccharides encounters multifaceted physical and chemical hurdles. Polysaccharides exhibit diverse physical and chemical attributes, while silicon nitride surfaces feature intricate charge distributions. Varied electron mobilities within distinct polysaccharide structures evoke diverse interactions [75]. Analyses based on blocking current amplitude, blocking duration, and event frequency have achieved notable differences in identifying analytes through nanopore detection methodologies. The extension of nanopore technology to anionic heparin sensing and detecting anionic OSCS impurities, facilitated by statistical threshold algorithms, has been accomplished. Solid-state silicon nitride nanopore sensors stand as reliable tools for heparin detection, the identification of enzymatic hydrolysis products, the direct differentiation of diverse polysaccharides, and hold promise for the clinical detection of chondroitin persulfate impurities in heparin (Figure 7a). Furthermore, it also analyzes the voltage polarity requirements and pH-dependent changes in the detection of polysaccharides in nanopore systems. The change in pH value of the solution can affect the charge sign and density of the analyte, thereby affecting the voltage polarity required for electrophoresis in a given direction. The chemical changes on the surface of nanopores dominate the effective mobility and its voltage polarity dependence. The frequency of events and voltage polarity behavior are consistent with the different physical and chemical properties of each analyte, and electrophoresis and electroosmosis occur simultaneously. In the negatively charged SiN_x_ pores at pH~7, anions A1 and A2 exhibit opposite directions of electroosmosis and electrophoresis, both producing signals in the electroosmotic direction. The electrophoretic force is greater on A2, which has a higher charge, compared to A1, reducing its detection frequency in the opposite electroosmotic direction. These findings offer novel experimental perspectives on the electrodynamic behavior in nanopores and the characterization of polysaccharides.

Facing the challenges inherent in analyzing glycosaminoglycan (GAG) samples with current analytical techniques, JongOne Im et al. innovatively combined solid-state nanopore single-molecule sensors with support vector machines (SVM) to develop a machine learning algorithm for GAG analysis. Their study underscored the potential of nanopore/SVM technology in the precise quantification and identification of GAG (Figure 7b) [76]. SVM, a supervised learning model that delineates distinct categories in a hyperdimensional space, has evolved as a crucial tool for processing single-molecule technical data [9,77]. This model has been extended to interpret solid-state nanopore data concerning GAG polysaccharide translocation. The outcomes affirm the capability of nanopore/SVM technology to discern heparin and chondroitin sulfate monodisperse fragments with exceptional accuracy (>90%), thereby enabling the detection of chondroitin sulfate impurities as low as 0.8% (*w*/*w*) in heparin samples. Furthermore, nanopore/SVM technology aptly distinguishes between unfractionated heparin and enoxaparin (a low-molecular-weight heparin).

Furthermore, Robert J. Linhardt et al. innovatively analyzed nanopore events as images and used deep neural network technology to identify polysaccharides. The initial success of solid nanopores in GAGs detection prompted Robert J. Linhardt et al. to employ nanopores for scrutinizing the controlled composition and sequence of heparan sulfate GAG chains [78]. Initially, a meticulously crafted set of heparan sulfate GAG chains, synthetically produced by chemical enzymes, was assembled with uniform length (around 40 disaccharide units), composition, and sequence. Each of the four fabricated GAG samples encompasses diverse disaccharide units, with each polysaccharide containing one to three sulfate groups assembled from a combination of four monosaccharide units. Analyzing polysaccharide nanopore events as pure images facilitated the application of Google’s deep neural network for image embedding, transforming images into feature vectors. Principal component analysis compressed these feature vectors for classification, revealing distinctive clusters denoting sample variations (Figure 7c). Furthermore, the SVM model precisely discerned GAG signal features in pure and mixed sample collections, effectively capturing sample representations at the disaccharide composition level. The findings suggest not only the analytical feasibility of disaccharide units within the synthesized GAGs but also the potential for sample classification based on monosaccharide units. Balme’s research group proposed a method to detect low concentrations of HEP and monitor the breakdown kinetics of heparin by heparinase using specialized conical nanopores coated with polylysine [79]. The binding of heparin with polylysine alters the surface charge within the nanopores, facilitating the detection of low heparin concentrations through rectification phenomena. Moreover, by leveraging the hindrance of heparinase hydrolysis activity by chondroitin sulfate, the detection of chondroitin persulfate was successfully accomplished.

### 4.2. Research on the Detection of Hyaluronic Acid Using Nanopore Technology

Hyaluronic acid (HA) is a polyanionic linear chain within the GAG family, characterized by alternating disaccharide repeat structures [80]. HA is prevalent in mammalian cells and tissues and plays crucial roles in various physiological functions, including tissue hydration, the extracellular matrix structure, innate immune regulation, and joint protection and lubrication [81]. Therefore, based on nanopore detection technology, direct detection methods are mainly used, with a focus on studying the molecular weight distribution and enzymatic hydrolysis performance of hyaluronic acid.

The exploration of oligosaccharide detection in the third part indicates that biological pores can be used for the detection of oligo hyaluronic acid. Therefore, biological nanopore technology can be used to study the enzymatic hydrolysis products of hyaluronic acid. Statistics can be used to analyze the distribution of functional groups, glycosidic linkage modes, and differences in isomers. The Bacri research group used the AeL nanopore to monitor the enzymatic degradation of long-polysaccharide-chain hyaluronic acid [82]. Further, Daniel’s group comparison of the detection performance of AeL and α-HL nanopores for glycosaminoglycans, hyaluronic acid oligosaccharides, and polysaccharides, found that α-HL nanopores with larger pore sizes can be used for the detection of hyaluronic acid polysaccharides, while AeL is more suitable for detecting oligosaccharides [71]. Then, they used wild-type AeL nanopores to detect glycosaminoglycans, and studied the size differentiation of tetra- to icosasaccharides from heparin, chondroitin sulfate, and dermatan sulfate, and demonstrated the ability to detect different contents and distributions of sulfate groups. They also detected differences in α/β anmerization and 1,4/1,3 osidic linkages were detected in heparin and hyaluronic acid, as well as a subtle difference between glucuronic/iduronic epimers in chondroitin and dermatan sulfate [83].

**Figure 8:**
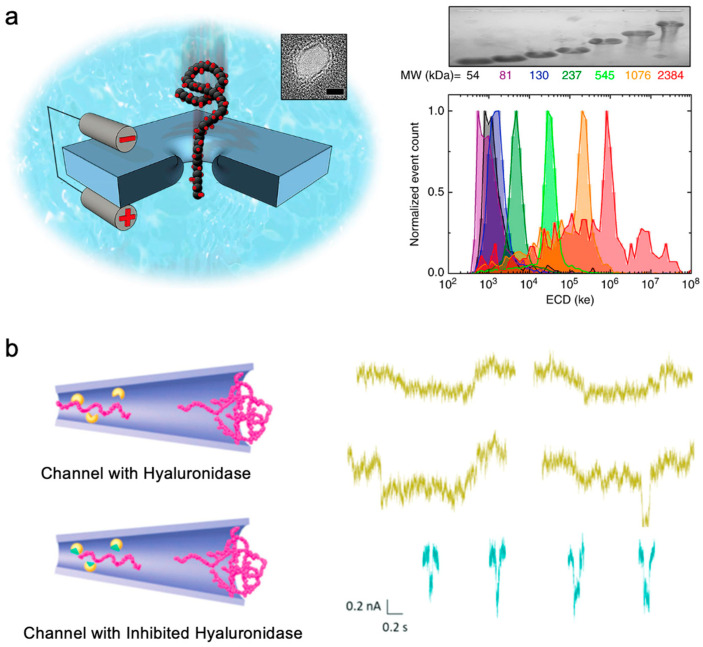
The detection of hyaluronic acid using solid-state nanopores. (**a**) A schematic diagram illustrating HA translocation through solid-state nanopores (left), alongside an ECD histogram for each molecular weight sample recorded at 200 mV, with colors corresponding to the molecular weight labels used in the gel image (right) [84]. (**b**) A schematic diagram showing the translocation of hyaluronic acid through glass conical nanopores modified with hyaluronidase on the tip side, along with an illustration of current blockage (top), with another schematic diagram displaying hyaluronic acid translocation through glass conical nanopores obstructed by hyaluronidase at the tip side, accompanied by an example of current blockade (bottom) [85].

Solid-state nanopore technology is an important means of detecting hyaluronic acid. Felipe Rivas et al. started from the perspective of standardized molecular conformation and used solid-state nanopore direct detection technology to determine the molecular weight distribution of hyaluronic acid based on the combined area of event amplitude and duration. The initial solid-state nanopore experiments conducted by Felipe Rivas et al. used a diverse mixture of HA with a wide molecular weight distribution isolated from Streptococcus suis fermentation, verifying the solid-state nanopores’ ability to differentiate HA [84]. To standardize the potential differences in molecular conformation, the combined area of event amplitude and duration was utilized. The results from nanopore analysis of HA populations with a narrow size distribution demonstrate that the molecular weight distribution can be inferred from the translocation signals of individual molecules (Figure 8a). Significantly, it was shown that the solid-state nanopore approach can delineate the size distribution of physiological HA obtained from the synovial fluid of an osteoarthritis horse model.

For the study of hyaluronidase degradation, unlike in biological nanopore research where hyaluronic acid and an enzyme are simultaneously placed in the detection solution, Balme et al. proposed a research scheme that modifies the enzyme at the entrance of the nanopore and directly observes degradation in real time. Balme’s research group explored the enzymatic degradation of high-molecular-weight HA within the range of 1.5 to 1.8 MDa at the single-molecule scale in specialized conical nanochannels [85]. For the first time, the direct translocation of high-molecular-weight hyaluronic acid was observed through these nanochannels utilizing resistance pulse technology. This discovery highlights the efficacy of this method as a valuable tool for predicting the migration of large flexible polyelectrolytes quantitatively or qualitatively in elongated conical nanochannels. By introducing a hyaluronidase inhibitor (quercetin) at the channel’s entrance, the group executed on-the-spot characterization of enzyme degradation reactions at the single-molecule level employing nanopore technology (Figure 8b), enabling the direct determination of enzymatic reaction duration at the individual molecule level.

### 4.3. Detection of Other Polysaccharide Using Nanopore Technology

**Figure 9:**
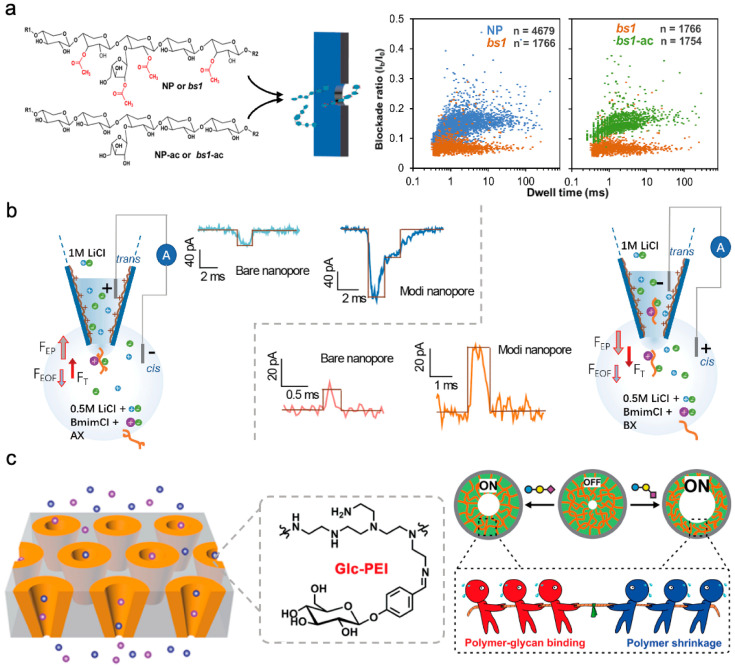
Structure recognition of plant and other polysaccharides using solid-state nanopore. (**a**) Differentiation of xylitol molecule acetylation and deacetylation using solid-state nanopores [89]. (**b**) Investigation of detection of homogeneous and heterogeneous xylans utilizing glass nanopores modified with polyethyleneimine (PEI) [90]. (**c**) Biomimetic nanochannel system incorporating the responsive polymer Glc-PEI for accurately identifying sialic polysaccharides [94].

Plant polysaccharides are an important class of polysaccharides. In various plant species, between 50% and 90% of cell wall constituents consist of polysaccharides [86,87]. Serving as a distinctive feature of plant cells, the cell wall fulfills essential functions in plant growth and development [88]. Our research group aims to investigate the structural analysis of plant polysaccharides using the direct detection of solid-state nanopores. Specifically, xylan and starch have been chosen as representative plant polysaccharides for the advancement of nanopore analysis technology. Initially, solid nanopores with pore diameters of around 3–4.6 nm were utilized to differentiate xylans modified with varying acetyl groups and varying surface charge, as illustrated in Figure 9a [89]. By analyzing the distribution of blocking amplitude and residence time under different voltage testing conditions, the group modification information of xylan was obtained. Ion current detection was then performed through nanopores on both unaltered natural xylans and highly acetylated rice mutant xylans. This analysis revealed distinct translocation behavior for polysaccharides with differing acetylation patterns, indicating potential applications in discriminating plant polysaccharides based on acetylation characteristics. Subsequent validation underscored acetylation as a critical factor influencing xylan molecule translocation behavior. Investigation into the translocation characteristics of diverse xylan types with varying surface charge attributes demonstrated the capability of solid-state nanopores to identify polysaccharide modifications and discern surface charge distributions.

Additionally, for water-insoluble polysaccharides, nanopore detection was carried out in combination with room temperature ionic liquids. Utilizing the strong polarity of room temperature ionic liquids to disrupt hydrogen bonds between water-insoluble polysaccharides, achieving single-molecule detection of water-insoluble polysaccharides based on nanopores, our research group delved into exploring homogeneous and heterogeneous xylan detection using PEI-modified glass nanopores [90]. Homogeneous xylan, lacking branched structures, exhibits near insolubility in water. In this study, the ionic liquid chlorinated 1-butyl-3-methylimidazole (BmimCl) was chosen as a solvent to dissolve both insoluble homogeneous and soluble heterogeneous xylans (Figure 9b). The nanopores were coated with polyethylene imine to enrich functional groups inside the nanopores, enhance interactions with xylan molecules, elevate the analytical capacity for polysaccharide architectures with nanopore technology, and lay the groundwork for the single-molecule detection of polysaccharides varying in water solubility. Furthermore, investigations extended to high-molecular-weight polysaccharides like starch were pursued [91], focusing on amylose and amylopectin. Despite starch’s abundance of hydrogen bonds, posing challenges in traditional solvent solubility for single-molecule studies, this research exhibited starch dissolution in BmimCl to establish a nanopore single-molecule detection technique tailored for starch. The discussion delved into the primary ions involved in starch dissolution by ionic liquids at a single-molecule scale, underscoring the utility of nanopore detection for probing the dissolution mechanisms of polysaccharide molecules.

For water-soluble neutral polysaccharides, our research group proposed a detection strategy for neutral polysaccharides by incorporating high-charge-density polymer molecules to boost the charge density of neutral polysaccharides [92]. In our study, dextran molecules served as the focal point. Dextran, consisting of glucose monosaccharide units, represents a significant polysaccharide with low molecular weight and has crucial medicinal applications [93,94]. Initially, we synthesized phenylboronic acid-modified polyethyleneimine molecules. Leveraging the bond formation between phenylboronic acid and hydroxyl groups on dextran, the charge density of pectin molecules was markedly heightened. This approach enabled the highly sensitive detection of dextran 70 through glass nanopores. Subsequent ECD analysis facilitated the differentiation between mixed dextran forms 20, 40, and 70. The outlined detection strategy, which augments the charge density of neutral molecules, not only enhances the detection sensitivity for other significant low-charge-density molecules utilizing nanopore technology but also underscores the system’s robust selectivity, sensitivity, and label-free attributes. These qualities indicate the promising utility of functional nanopore platforms in glycomics and the advancement of polysaccharide-related devices.

For high-molecular-weight polysaccharide molecules, ion current detection based on conical nanopores is also an important detection strategy. Salivary glycans attached to cell surfaces play crucial roles in mediating diverse cellular processes, notably different linker isomers that can instigate various biological events and signify distinct cancer types. Therefore, the accurate determination of sialic glycans is imperative [95,96]. Minmin Li and colleagues introduced a biomimetic nanochannel system incorporating a responsive polymer, polyethyleneimine-g-glucan (Glc-PEI), for the precise identification of sialic polysaccharides [97]. Studies have highlighted that the interaction between Glc-PEI and polysaccharides, and the interplay of Glc-PEI polysaccharide binding with Glc-PEI contraction, result in variations in Glc-PEI’s contraction level, culminating in an enlargement of nanopore dimensions and enhanced ionic conductivity. The presence of 2-6 polysaccharides induced a marked alteration in ion current, with Glc-PEI-modified PET demonstrating distinctive discrimination between 2-6 polysaccharides and 2-3 polysaccharides (Figure 9c).

## 5. Detection of Glycosylation Biomolecules

Glycosylation, facilitated by enzymes, involves attaching saccharide molecules to proteins or lipids. Through glycosyltransferases, saccharide molecules are transferred to proteins, forming glycosidic bonds with amino acid residues [98]. The post-translational modification (PTM) of glycosylated proteins significantly influences cellular processes and contributes to the pathogenic mechanisms of numerous diseases [99]. As such, identifying PTMs is essential for comprehending intricate cellular processes and disease pathology [100]. Moreover, glycoconjugates like glycoproteins and glycolipids are prevalent in cells, on cell surfaces, and in the extracellular milieu. Glycoproteins serve as critical markers in various diseases and clinical diagnostics, with an expanding pool of glycoproteins considered potential biomarkers [101]. Investigation into glycosylation biomolecules using nanopore technology encompasses the examination of glycosylation sites and structures, alongside the high-sensitivity detection of glycoprotein molecules. The exploration of glycosylation sites and structures primarily integrates resistance pulse detection technology in biological nanopores, while glycoprotein recognition relies on saccharide molecule identification by boric acid-modified solid-state nanopores. Furthermore, our research group has pioneered a detection and analysis methodology for lipopolysaccharides founded on solid-state nanopores.

### 5.1. Research on Glycosylation Peptide Detection Based on Biological Nanopore Technology

**Figure 10:**
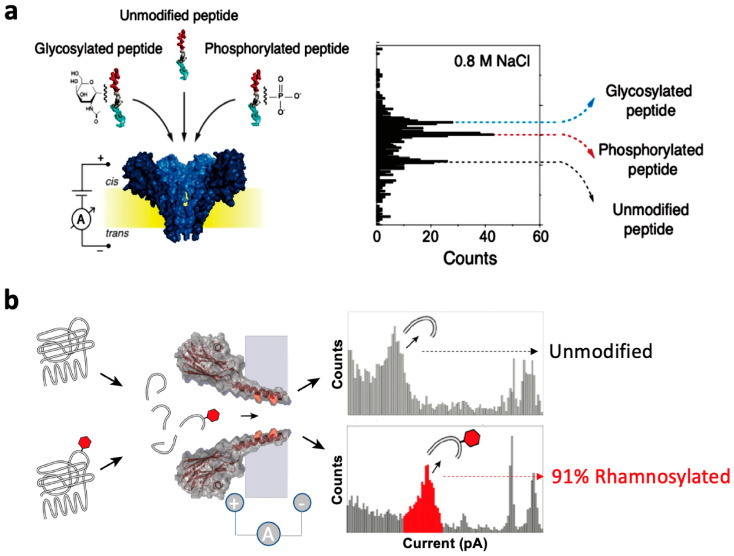
Glycosylation peptide detection based on biological nanopore. (**a**) The detection of glycosylated and phosphorylated peptides at the single-molecule level using the FraC biological nanopore from crustacean toxin is detailed [102]. Additionally, (**b**) phenylalanine-modified crustacean toxin FraC nanopores differentiate hydrophilic peptides from glycosylated peptides [103].

Due to the small structure of glycosylated peptide sites, the key to detecting glycosylated peptide sites lies in controlling the selection of suitable nanopores and slowing down via the rate in order to effectively recognize via signals. There are two main strategies: one is to perform opposite charge modification on both ends of the glycosylated peptide fragments to slow down the translocation velocity. Another approach is to engineer the nanopores to increase the interaction between glycosylated peptide fragments and the nanopores, thereby delaying the translocation time. Laura Restrepo-Pérez and colleagues introduced a method for the single-molecule level detection of PTM utilizing the Fragaceatoxin C (FraC) nanopore [102] (Figure 10a). Utilizing a bipolar model peptide where two charged regions are linked by a flexible glycine and serine sequence, the peptide halts within the nanopore, facilitating the highly sensitive detection of specific regions. Studies have revealed the distinguishable detection of glycosylated and phosphorylated peptides from unmodified peptides based on variations in relative current blockage and residence time during nanopore traversal. Notably, the groundbreaking differentiation between glycosylated and phosphorylated peptides in a label-free manner marks a crucial stride towards single-molecule and label-free identification of protein variants. Giovanni Maglia’s research team proposed the use of a high-ionic-strength crustacean toxin FraC nanopore, featuring low pH conditions and a phenylalanine mutant at its constriction site, for discerning hydrophilic peptides and discriminating monosaccharide from disaccharide peptides [103] (Figure 10b). The interaction between phenylalanine and positively charged peptides (via cation–π interaction) and carbohydrates (via π stacking interaction) prolongs the residence time of glycosylated peptides within the nanopores. This approach accurately identifies and quantifies glycosylation levels on cyclic peptides and naturally hydrolyzed EF-P protein peptides, showcasing the capacity of this nanopore technique in discerning and quantifying glycosylation on ubiquitous proteins has promising potential in disease diagnosis and cell biology.

### 5.2. Research on Glycoprotein Detection Based on Solid-State Nanopore Technology

**Figure 11:**
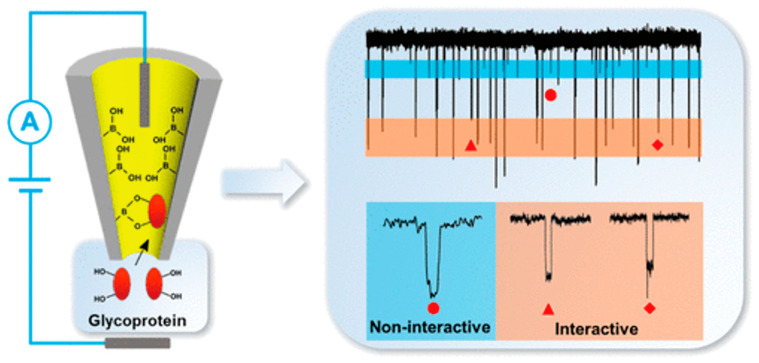
Schematic diagram of label-free detection of single-molecule glycoproteins based on boronic ester affinity interaction using thiol phenylboronic acid-modified glass conical nanopores, and current time curve of IgG in 4-MPBA-modified glass conical nanopores [104].

Due to the specific affinity interaction between boronic acid and glycoproteins, researchers have developed numerous strategies for glycoprotein detection using boronic acid-modified affinity materials. These nanopore-based platforms for glycoprotein sensing can find applications in the biomedical field for characterizing carbohydrate components on cell surfaces. Yongxin Li’s research group proposed employing nanopore-based single-entity electrochemistry and sugar borate affinity for label-free monitoring of single-molecule glycoprotein resistance pulse detection based on nanopore technology for label-free monitoring of single-molecule glycoproteins using monomer electrochemistry and affinity for sugar borate [104] (Figure 11). They adapted an ethanol solution of 4-mercaptophenylboronic acid into glass conical nanopores. Studies have revealed that the multivalent enhancement effect within confined spaces results in a higher sugar boronic acid affinity force within these spaces than at a global level. The sensor was effectively utilized to detect IgG in the urine of patients with idiopathic membranous nephropathy, achieving an almost 100% recovery rate using the spiked method. Nguyen and colleagues successfully recognized and detected sugar-modified protein molecules through reversible binding between boronic acid and glycoproteins, leading to notable changes in ion flux in nanochannels [105]. These findings underscore the potential application value of functional nanopore analysis tools for real sample glycoprotein detection.

### 5.3. Research on Lipopolysaccharide Detection Based on Solid-State Nanopore Technology

Lipopolysaccharide (LPS) constitutes the principal component of the cell membrane in all Gram-negative bacteria [106]. The key distinctions in LPS across various bacterial species lie in the O antigen and polysaccharide fraction [107]. Our research group has developed a nanopore-based resistive sensing direct detection platform combining proximity analysis of blockage signals to distinguish LPS from diverse bacterial species and varying serotypes of the same bacterium. The study involved LPS nanopore detection from six bacteria, namely Escherichia coli, Escherichia coli B5, Salmonella, Klebsiella pneumoniae, Escherichia coli J5, and Pseudomonas aeruginosa. Variations exist in the molecular structure of LPS among different bacteria, particularly in the lipopolysaccharide A tail count across bacterial strains. Proximity analysis of the current blockage signal is observed via the indicated significant differences in the nanopore transport behavior of LPS among distinct bacterial species. While LPS serotypes within the same bacterium typically exhibit structural resemblance, the analysis of residence time, amplitude, and ΔI/I_o_ suggests discernible differences among the three LPS serotypes. These results suggest the potential of nanopore technology in discriminating LPS serotypes, underscoring the promising utility of nanopore sensing platforms in clinical and environmental biomarker detection, offering a valuable screening tool for the early detection of water and medical product contamination.

## 6. Summary and Prospect

This article presents a comprehensive review of the advancements in saccharide molecule detection leveraging nanopore technology, delves into its application for detecting monosaccharides, oligosaccharides, and polysaccharides, and anticipates its potential future applications in scientific research and clinical diagnostics. Saccharide molecules are vital components of living organisms, playing a critical role in maintaining cellular structure and function. However, the complex and diverse structures of saccharide molecules pose challenges for traditional detection methods, leading to limitations such as low resolution and the inability to achieve single-molecule detection. Nanopore technology emerges as a novel single-molecule detection method that senses the passage of molecules through nanoscale pore channels, facilitating molecule recognition and analysis. With benefits like high sensitivity, label-free operation, and real-time monitoring, nanopore technology is well-suited for detecting intricate biomolecules.

We outline various nanopore detection approaches utilized for distinct saccharide molecules. For instance, monosaccharide detection involves achieving high sensitivity, notably for glucose, through the resistance pulse method using biological nanopores or ion rectification detection in solid-state nanopore technology. Oligosaccharide detection relies on single-molecule detection technology for biological nanopores to identify different polymerization degrees and distinguish glycosidic bond connection modes by analyzing current pulse blockage signals. Similarly, polysaccharide detection employs solid-state nanopore technology to identify polysaccharides like heparin and hyaluronic acid, analyzing resistance pulse signals to differentiate various polysaccharides and offer a fresh approach to polysaccharide structure detection. It can be seen that solid-state nanopores and biological nanopores have their own advantages and disadvantages in the detection of saccharide molecules. Biological nanopores can provide corresponding structural information for the detection of small molecular weight sugar molecules such as monosaccharides and oligosaccharides, but cannot be used for the detection of nonlinear polysaccharide molecules. Solid-state nanopores are suitable for the detection of high-molecular-weight polysaccharide molecules, and, combined with algorithms, corresponding structural information can be obtained. The performance of biological nanopores in detecting the structural information of sugar molecules is superior to that of solid-state nanopores. Solid-state nanopores have significant advantages as biosensors for the quantitative detection of polysaccharides due to their good stability. Here, we summarize the advantages and disadvantages of various nanopore detection techniques for detecting carbohydrate molecules (Table 1).

Nanopore detection technology exhibits promising applications in saccharide molecule detection. For example, it shows significant potential in diagnosing diseases related to saccharides like diabetes and kidney diseases. By monitoring the impact of drugs on saccharide molecule structures, nanopore technology aids in new drug development and screening. It can also evolve into being used for biosensors for environmental monitoring and food safety applications by utilizing the interaction between saccharide molecules and specific receptors. Despite its potential, nanopore technology faces challenges such as enhancing detection selectivity, expanding detection range, and improving data analysis capabilities. Future research directions include developing innovative materials to enhance pore channel stability and selectivity, utilizing machine learning and artificial intelligence for improved signal processing accuracy and efficiency, and integrating nanopore technology with other detection methods to establish a multifunctional integrated detection platform. For example, solid-state nanopores have the advantages of good environmental adaptability, customizable nanopore size, and large-scale integration. However, the reproducibility of solid-state nanopore preparation is poor. Therefore, the direct synthesis of nanoscale pores is expected to achieve precise control of the pore size at the atomic level. At present, research has focused on the synthesis of large-diameter cyclic molecules [108,109] and nanoscale two-dimensional pore nanomaterials [110,111], which have been preliminarily applied in the field of nanopore detection. We believe that such nanoporous materials prepared directly through chemical synthesis methods can be directly integrated into nanopore detection platforms in the future. These efforts aim to advance the clinical utility of nanopore technology, transitioning from laboratory to clinical practice. With ongoing technological advancements, nanopore technology is expected to have a more substantial role in future scientific research and medical health fields.

## Figures and Tables

**Table 1:** The main nanopore detection strategies and saccharide molecule types are reviewed.

Nanopore Types		Sensing Strategy		Analyte Types	Pros	Cons	Ref.
Biological nanopore	ClyA	Resistive pulse sensing	Protein assist	Monosaccharide	Specific recognition	Cannot determine the structure	[46,47]
				Oligosaccharides	Identifies the bond types	Not universal	[63]
	α-HL, MspA		Engineered nanopore	Monosaccharides	Identifies multiple polysaccharides	Nanopore needs engineering	[49,51]
	α-HL			Oligosaccharides			[68]
	AeL		Incorporate labels	Oligosaccharides	Oligosaccharides with low charge density	Analytes need to be labeled	[65]
	AeL, α-HL		Direct detection	Oligo/Polysaccharides	Simple and direct	For high-charge linear molecules	[69,70,71]
	CymA					Needs specific membrane pore proteins	[66]
	FraC			Glycosylation peptide	Simple and direct		[102,103]
Solid-state nanopore	PET, Glass	Ion rectification	Charge change	Mono/Polysaccharides	High sensitivity	Nanopore modification, unable to perform structural analysis	[56,57,59,79]
	Glass		Structure change	Monosaccharides			[61]
	CN	Resistive pulse sensing	Direct detection	Oligosaccharides	Directly distinguishable structure	Multi- nanopore structure	[72]
	SiN_x_			Polysaccharides Lipopolysaccharide		Multi- condition testing	[89,106]
	SiN_x_		Detection through algorithms		Direct detection	Suitable for high molecular weight, charge and linear molecules	[75,76,78,84]
	Glass		Enzyme modification		Real-time reaction monitoring		[85]
			Chemical modification	Glycoprotein	High sensitivity	Nanopore modification	[104]
	SiN_x_, Glass		Detection via ionic liquids	Poly- saccharide	Polysaccharides with low charge density	Electrolyte contains multiple solvents	[90,91]
	Glass		Chemical molecules assistant			Introduction of other molecules	[96]
